# Second-Degree Interatrial Block in Hemodialysis Patients

**DOI:** 10.1155/2015/468493

**Published:** 2015-02-10

**Authors:** Andres Enriquez, Marco Marano, Anna D'Amato, Antoni Bayes de Luna, Adrian Baranchuk

**Affiliations:** ^1^Division of Cardiology, Department of Medicine, Queen's University, Kingston, ON, Canada; ^2^Hemodialysis Unit, Maria Rosaria Clinic, Pompeii, Italy; ^3^Hospital de la Santa Creu i Sant Pau, Cardiovascular Research Center, CSIC-ICCC, Barcelona, Spain

## Abstract

Interatrial conduction delays manifest as a prolonged P-wave duration on surface ECG and the term interatrial block (IAB) has been coined. They are usually fixed, but cases of intermittent IAB have been described, suggesting functional conduction block at the Bachmann bundle region. We report 2 cases of patients on chronic hemodialysis therapy presenting with intermittent IAB.

## 1. Introduction

Interatrial block (IAB), defined as a conduction delay between the right and left atrium, is manifested on the electrocardiogram (ECG) as a prolonged P-wave duration and can be classified as partial (P-wave ≥ 120 ms) or advanced (P-wave ≥ 120 with biphasic [±] morphology in the inferior leads) [1]. However, IAB may also be intermittent [2] and the term “second-degree IAB” has been proposed [1]. We report 2 cases of patients on chronic hemodialysis therapy presenting with second-degree IAB.

## 2. Patient 1

**Figure 1:**
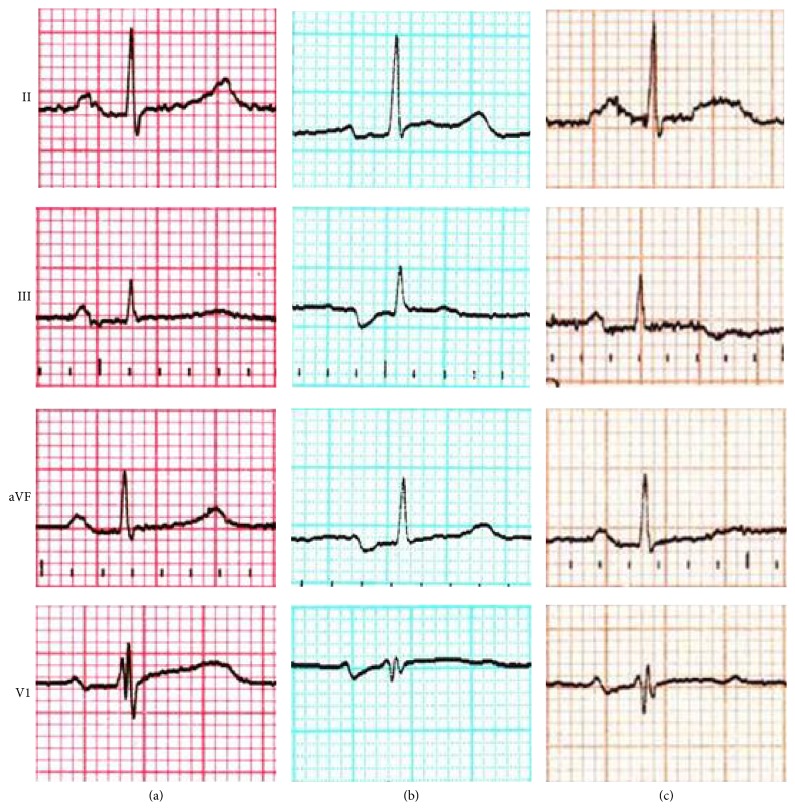
(a) 4-lead ECG 1 month prior to the episode. Normal P-wave. (b) 4-lead ECG during the episode. Note advanced IAB without changes in lead V1, confirming this is not an ectopic rhythm. (c) 4-lead ECG after the episode. Changes have disappeared.

First case is an 81-year-old female with end-stage renal disease of unknown etiology who presented to her hemodialysis session complaining of weakness and nausea. No prior history of atrial fibrillation (AF) was present. The previous day, after taking 50 mg of tramadol at bedtime for bone pain, she experienced malaise and vomiting. At the hemodialysis unit next morning, she was still feeling nauseous. Her blood pressure was low (90/60 mmHg) and interdialytic weight gain was only 0.8 Kg. An ECG recorded in the context of vomiting showed a prolonged P-wave (120 ms) with a prominent negative terminal component in the inferior leads, compatible with a pattern of advanced IAB (Figure 1(b)). In contrast, a resting ECG performed 1 month earlier had shown sinus rhythm with normal P-wave duration and PR interval (Figure 1(a)). The similar P-wave morphology in lead V1 suggests that both tracings correspond to sinus rhythm rather than an ectopic rhythm. Blood gas analysis revealed normal acid-base status and electrolytes. Predialysis serum total concentration of calcium was 9.4 mg/dL and of potassium was 4.8 mEq/L. Predialysis blood gas analysis showed ionized calcium of 1.14 mmol/L and potassium of 4.4 mmol/L. Bicarbonate-dialysis was performed. The session was uneventful, with 1 L fluid removal. Potassium and calcium dialysate concentration were 3.0 mEq/L and 1.5 mEq/L, respectively. Two days later, a repeated ECG showed resolution of the advanced IAB (Figure 1(c)).

## 3. Patient 2

**Figure 2:**
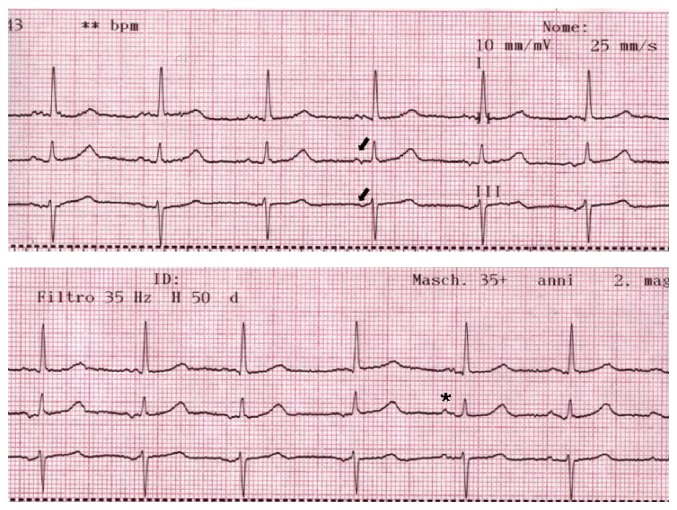
Continuous ECG tracing (leads I, II, and III), showing a prolonged P-wave duration, with transient development of a negative component in II and III (arrow), which resolves after 7 beats (asterisk).

Second case is an 86-year-old lady with hypertensive end-stage renal disease who had been on hemodialysis therapy in our center since 2006. Her past medical story was significant for long-standing hypertension (40-year duration), ischemic heart disease with myocardial infarctions in 2006 and 2012, a transient ischemic attack in 2012, osteoporosis with low-normal parathyroid hormone (adynamic bone disease), and no prior history of AF. Her medications list included Clopidogrel 75 mg, Atorvastatin 40 mg, Bisoprolol 2.5 mg, Amlodipine 10 mg, Omeprazole 20 mg, and Epoetin 4000 units. During a routine hemodialysis session she referred to mild dyspepsia. Predialysis serum total concentration of calcium was 8.4 mg/dL and of potassium was 5.7 mEq/L. Predialysis blood gas analysis showed ionized calcium of 1.06 mmol/L and potassium of 5.1 mmol/L. The dialysis was 240 minutes' duration, with 1.8 L fluid removal, bicarbonate 35.0 mmol/L, K 3.0 mmol/L, and Ca 1.5 mmol/L. Soon after dialysis treatment, she complained of heartburn, bloating, nausea, and vomiting. Blood pressure, arterial blood gas analysis, and amylase levels were all normal. A 12-lead ECG showed partial IAB (P-wave 130 ms), with transient development of a terminal negative component in the inferior leads, which resolved after a few beats without significant change in heart rate (leads I, II, and III shown in Figure 2).

IAB is a prevalent condition, especially in patients with structural heart disease, and is frequently associated with atrial arrhythmias, such as AF and flutter [3–6]. This association is particularly strong for patients with advanced IAB. This electrocardiographic pattern is consequence of marked conduction delay at the Bachman region and retrograde (caudocranial) activation of the left atrium mainly through the coronary sinus, which explains the negative terminal deflection of the P-wave in II, III, and aVF [7, 8]. Advanced IAB leads to heterogeneous electrical activation, dispersion of refractoriness, and left atrial mechanical dysfunction, all factors that predispose to the genesis of atrial arrhythmias [9].

The present cases show that, similarly to conduction blocks at the level of the atrioventricular junction, sinoatrial junction, or the ventricles, IAB may occur transiently and intermittently. Intermittent IAB can occur with every other sinus beats or without a recognizable pattern and the mechanism is unclear. The most likely explanation is a functional block in one or more of the specialized interatrial tracts [2]. Positional changes of the atria can be postulated, but this is unlikely since previous reports have shown that patients with intermittent IAB may develop fixed IAB over time [2]. Transient IAB has also been described in the context of decompensated heart failure, which has been attributed to atrial strain and is reverted to by diuretic therapy [10]. The 2 cases presented here were observed in the context of emesis. It is possible that some degree of autonomic or acute electrolytic disturbance may play a role in the development of functional block.
